# A bibliometric analysis of progress and trends in pediatric glioma research

**DOI:** 10.1007/s12672-026-04445-y

**Published:** 2026-01-28

**Authors:** Mengping Wang, Chenchen Wu, Zhen Zhang, Xiwei Li, Junyi Liao, Mingyue Zhang, Enlin Jian, Xiaoping Yu, Yanfeng Zhu, Peiling Cai

**Affiliations:** 1 School of Preclinical Medicine, Chengdu University, No. 2025, Chengluo Avenue, Chengdu, 610106 Sichuan China; 2 Clinical Medical College & Affiliated Hospital of Chengdu University, Chengdu, 610106 Sichuan China; 3 School of Public Health, Chengdu Medical College, No. 783, Xindu Avenue, Chengdu, 610500 Sichuan China

**Keywords:** Children, Glioma, Bibliometric analysis, Hotspots, CiteSpace

## Abstract

This bibliometric study analyzes 4,861 publications on pediatric gliomas (PGs) from the Web of Science Core Collection (WoSCC) to map the field’s 60-year research trajectory. Despite recent scientific advances, the analysis of long-term trends, interdisciplinary dynamics, and comprehensive collaboration networks is still insufficient. USA leads in output, with St. Jude Children’s Research Hospital as the top institution and Childs Nervous System as the dominant journal. Pioneering authors including Bouffet Eric, Gutmann David H., Packer Roger, and Grill Jacques established foundational work guiding subsequent research. Keyword analysis identifies “Glioma” and “Children” as central themes. This study integrates the constantly evolving research frontiers and provides important insights for scholars’ future research.

## Introduction

Among childhood malignant tumors, brain and spinal cord tumors account for 20% of the cases. Their fatality rate is second only to that of leukemia. Brain and spinal cord tumors are the second leading cause of death related to childhood tumors [1]. Glioma is a primary brain tumor that originates from neural stem cells or neural precursor cells and has oncogenic mutations [2], and gliomas are graded by the WHO (World Health Organization) Central Nervous System (CNS) Tumor Classification into grades 1–4 based on histopathology and molecular features, spanning from benign to highly malignant forms [3]. Among various types of brain tumors in children, pediatric gliomas (PGs) account for approximately 40 to 50% [4, 5]. Recent genomic and epigenomic studies have revealed that PGs have distinct molecular characteristics and biological manifestations compared to adult gliomas. For instance, common BRAF fusion genes and H3K27M mutations are present in PGs [3, 6]. Compared with adult gliomas, PGs have a lower degree of malignancy; however, the prognosis of these tumors largely depends on their anatomical location [7]. When it comes to risk factors, the impact of environmental exposure is significant. If parents are exposed to solvents, polycyclic aromatic hydrocarbons (PAHs) at work, or are employed in the chemical industry, the risk of their children developing PGs will increase significantly [8, 9]. Furthermore, the use of pesticides by pregnant women during pregnancy may also pose a potential threat [10]. Additionally, genetic syndromes such as tuberous sclerosis complex (TSC) and neurofibromatosis type 1 (NF1) have been confirmed as definite genetic susceptibility factors. They are respectively closely related to optic pathway gliomas and ventricular subependymal giant cell astrocytomas [7, 11]. It is worth noting that during pregnancy, taking in sufficient vitamins, especially folic acid, vitamin C and vitamin E, may have a protective effect by acting as antioxidants and promoting cell differentiation [12, 13]. Based on the natural clinical course of untreated cases, PGs can be broadly classified into two categories: LGGs (low-grade gliomas, WHO grade I or II, with most of them belonging to mixed glioneuronal tumors) and HGGs (high-grade gliomas, WHO grade III or IV). However, the applicability of this classification system is not yet fully clear in some newly discovered tumor variants [14]. These findings have reshaped diagnostic and therapeutic approaches [3]. Since the inception of WHO’s CNS tumor classification in 1961 [15], PGs research has transitioned from a histopathology-centric paradigm to an era of integrated molecular characterization. The fifth edition of the WHO classification released in 2021 represents a significant shift [16]. It established an independent classification system for PGs and prioritized the use of molecular markers such as isocitrate dehydrogenase (IDH), H3K27M, and BRAF to guide precision medical strategies [17–19].

Packer et al. in their review of studies on NF1-associated gliomas in children emphasized the following key points [20]. Firstly, the main driving factor for childhood NF1-related LGGs is the loss of both alleles of the NF1 gene [21]. Secondly, it is usually not recommended to make a diagnosis through biopsy, and regular visual screening examinations are suggested instead [22]. Third, the first-line treatment consists of carboplatin and vincristine chemotherapy [23], while radiotherapy should be avoided. Fourth, in targeted therapy, MEK inhibitors (e.g., selumetinib) have demonstrated significant efficacy and emerged as a key therapeutic option [24]. Fifth, the primary treatment goal is the preservation of functional outcomes, particularly vision [20]. Future research should explore the potential of immunotherapy and the long-term effects of targeted therapies. In Kasper’s review, the author proposed that mutations in histone H3, particularly the H3K27M mutation, are the key driving factor for pediatric high-grade gliomas (pHGGs) [25]. These mutations will bind to the Polycomb repressive complex 2 (PRC2), and continuously inhibit the activity of PRC2 in this way to exhibit a dominant negative effect [19], ultimately causing the loss of H3K27me3 throughout the entire genome [26]. This mechanism, in conjunction with the absence of p53 and the activation of PDGFRA in neural precursor cells (such as brainstem OPC-like cells) during specific developmental stages, drives tumor formation, while determining the midline positioning and blocking the differentiation process [26–28]. In a 2021 review published in Neuro-Oncology, Milde et al. proposed that the continuous activation of the mitogen-activated protein kinase (MAPK) signaling pathway is the core pathological mechanism of pediatric low-grade gliomas (pLGGs), and this is particularly evident in pilocytic astrocytoma (PA) [29]. However, the emergence and maintenance of these tumors rely on supportive signals derived from the specific tumor microenvironment (TME). Some of these signals come from microglia, while others come from T lymphocytes [30–32]. Moreover, these supportive signals are also influenced by the Oncogene-Induced Senescence mechanism [33, 34]. Understanding the necessity of the synergy between driver gene mutations and microenvironmental support is crucial for elucidating the inert growth and spontaneous regression of pLGGs [35–37], and provides a basis for treatment strategies such as MEK inhibitors [29, 38]. In the review written by Fangusaro et al. on pLGGs, the authors presented several key points: the most common CNS tumor in children is pLGGs. Its main characteristic lies in the abnormal activation of the MAPK/ERK and mTOR signaling pathways. Situations such as BRAF fusion or mutation, and FGFR1 alterations fall under this category [5]. This characteristic of the molecules has led to their reclassification as chronic diseases, and the treatment goals have also changed accordingly. For instance, the focus has shifted to functional outcomes, such as vision preservation and quality of life, rather than merely striving to prolong survival [39]. Molecularly targeted therapies, such as MEK inhibitors (e.g., selumetinib [24]) and BRAF/MEK inhibitor combinations (e.g., dabrafenib plus trametinib [40]), have significantly transformed the treatment landscape. However, key challenges still remain, including drug resistance, tumor recurrence after treatment interruption, and relapse [41]. The assessment of treatment response was conducted using the pediatric-specific RAPNO imaging criteria, with a particular focus on functional endpoints such as visual outcomes [42, 43]. Future research will need to address three crucial issues: first, to establish effective preclinical models; second, to standardize the design of early clinical trials; and third, to understand the underlying causes of drug resistance, rebound and recurrence [44]. Research in the PGs field has made considerable progress, but currently there is a lack of systematic and comprehensive literature reviews. This makes it difficult to integrate the theoretical framework and apply it in practice.

Bibliometrics is a method that employs numerical and statistical techniques for quantitative analysis. It relies on mathematical and statistical modeling to visualize shifts within specific knowledge domains, thereby clearly identifying core research elements such as key scholars, influential research nations, and impactful journals [45]. This method not only traces the evolution trajectory of the discipline, clarifies the development path of research, but also outlines the current research focus and the future development direction [46]. Although the number of research literature related to PGs has been increasing, there is still a lack of long-term and systematic bibliometric studies in the field of PGs. If researchers rely only on non-long-term research, the analysis results will be easily influenced by various factors, and eventually lead to incorrect or one-sided judgments on the prevailing trends. Only by conducting long-term tracking can the true existing trends be identified, and the limited resources can be allocated to the most valuable directions, avoiding scholars’ continuous attention to those “useful in the short term but useless in the long term” projects. We used the method of bibliometrics to conduct a systematic review and evaluation of relevant academic literature. This approach enables researchers to objectively track the key development trends in this field, clearly identifying the current main research directions, frequently discussed topics, and cutting-edge hotspots. Through this analysis, the aim is to provide some practical references for future scholars studying PGs. Therefore, this study utilized the literature data from the Web of Science Core Collection (WoSCC) from 1961 to 2024. Using bibliometric methods, it conducted quantitative analysis and visual presentation, aiming to understand the overall research landscape in the PGs field and identify emerging academic trends.

## Materials and methods

### Data source

Web of Science, due to its comprehensive citation index and diverse analytical indicators, can help us identify the research hotspots and trends in a particular field [47]. This study obtained the published PGs literature data from the WoSCC database for conducting bibliometric analysis. To minimize the bias caused by data updates, all the retrieval, extraction and download were completed within one day. The literature was limited to articles and reviews, and only in English. Since the data was directly obtained from the database without the need for additional animal experiments, no ethical approval was required.

### Data acquisition

**Fig. 1:**
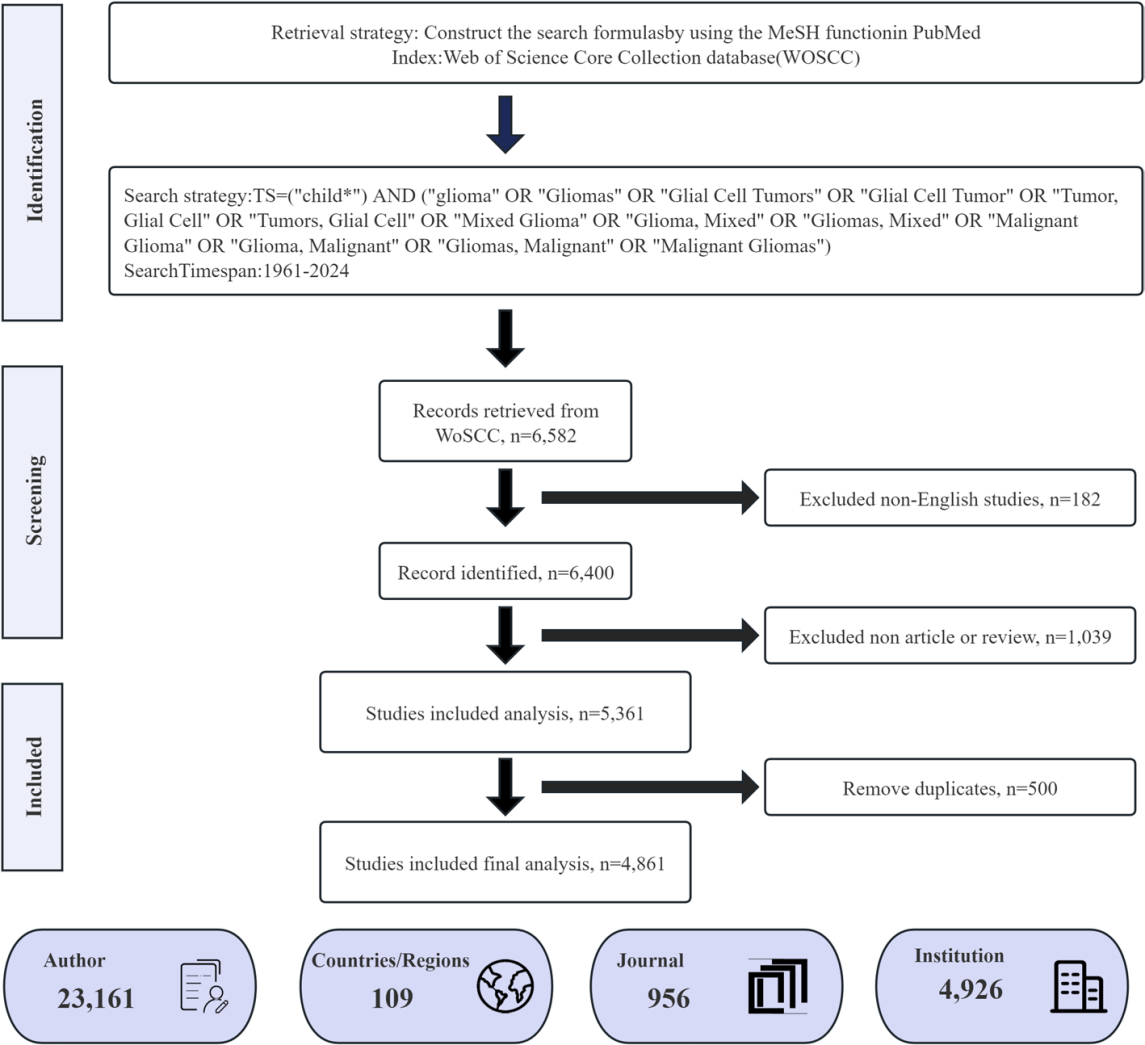
Flow chart of the literature selection process

A comprehensive search was conducted in the WoSCC database from January 1, 1900 to December 31, 2024. This search was completed on February 5, 2025. The full-text records of the retrieved literature were later downloaded in plain text format. The query strategy employed a set of keywords and Boolean operators: TS=(“child*”) AND (“glioma” OR “Gliomas” OR “Glial Cell Tumors” OR “Glial Cell Tumor” OR “Tumor, Glial Cell” OR “Tumors, Glial Cell” OR “Mixed Glioma” OR “Glioma, Mixed” OR “Gliomas, Mixed” OR “Malignant Glioma” OR “Glioma, Malignant” OR “Gliomas, Malignant” OR “Malignant Gliomas”). Due to the limited number of available publications in 2025, this search only covered English articles and reviews published up to 2024 (ignoring the early visit records), excluding Letters, News Items, Early Access, Meetings, Meeting Abstracts, Corrections, Reprints, Proceedings Papers, Book Chapters, Editorial Materials, Retracted Publications. We manually performed duplicate removal for each individual document. Figure 1 shows the selection and analysis process used when collecting the literature.

### Bibliometric analysis

This study employed a multimodal bibliometric analysis approach: first, we selected VOSviewer 1.6.20 and Prism 10.1.2 as the two powerful tools. Through these means, we not only constructed a keyword co-occurrence network that reveals the correlations of research hotspots, but also meticulously examined the footprints of collaborative efforts - including the institution collaboration network (including institutions with a publication volume of ≥ 20), the author collaboration network (including authors with a publication volume of ≥ 15), and the country/region collaboration network (with a threshold set at 1). Concurrently, CiteSpace 6.4.1 and R-bibliometrix 4.4.1 tools were integrated to generate keyword co-occurrence mapping and research frontier detection, respectively, thereby establishing a multidimensional analytical framework. Specific parameter settings: the time span was set from January 1961 to December 2024. All analytical data were derived from publicly available databases, with no ethical review requirements involved.

## Results

### Analysis of publications

**Fig. 2:**
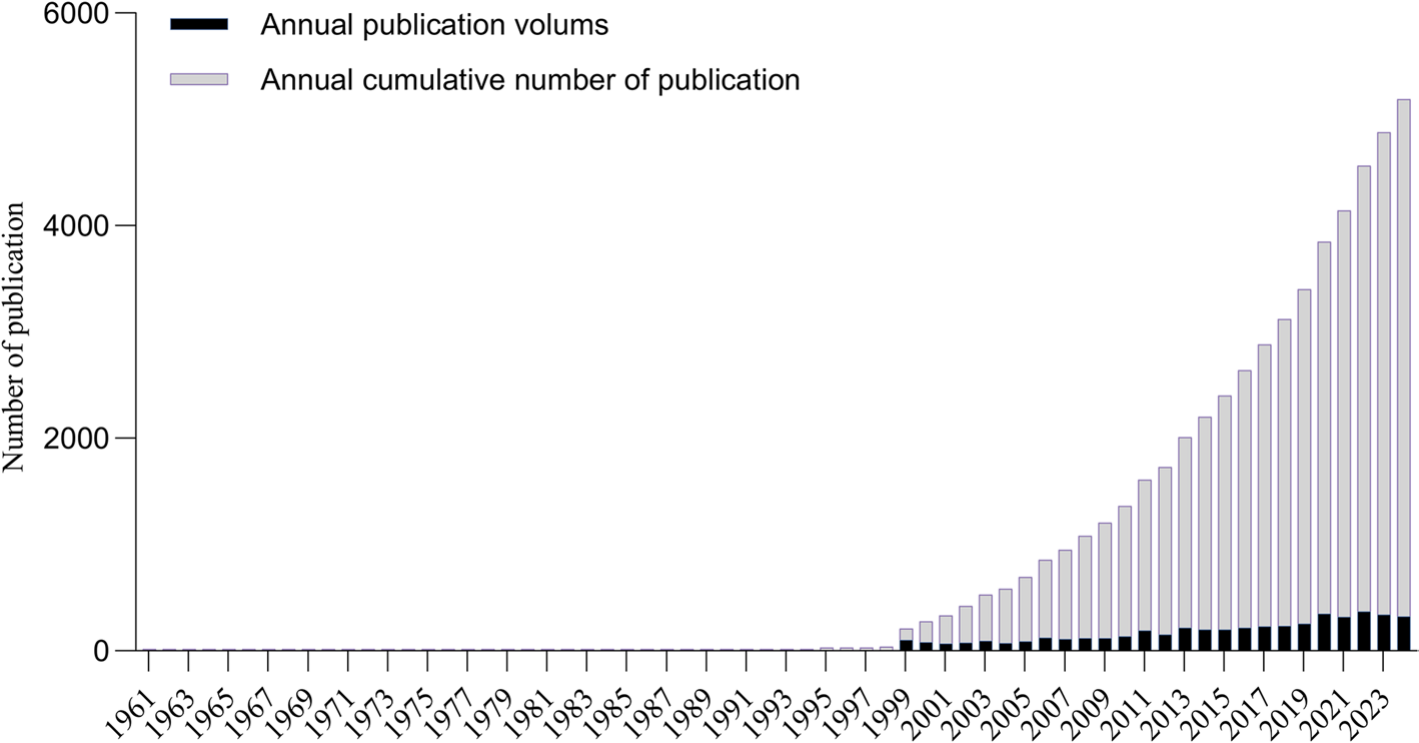
The cumulative and annual number of publications from 1961 to 2024. The Y-axis represents the cumulative number of published papers, and the X-axis represents the year

We can observe the annual scientific output of PGs research from 1961 to 2024 in Fig. 2. Research on PGs received minimal attention before 1999 but began rapid growth thereafter. Over the past decade, the number of academic papers published has been steadily increasing. From 2015 to 2020, the annual output of papers in this field increased by approximately 1.7 times. Although there were slight fluctuations in the number of publications from 2020 to 2024, the overall trend remained stable. In 2022, 372 papers were published, which was the peak during this period and also the year with the most prominent research output in this field. Since 2022, the number of published papers in this field has remained at a high level, which clearly demonstrates the significance of PGs research. The increase in the number of published papers over the past two decades may be related to the rising incidence of PGs [48], as well as the emergence of new therapies such as immunotherapy and targeted therapy - these breakthroughs not only opened up new treatment directions but also increased the enthusiasm for research [49–51].

### Analysis of countries/regions

**Fig. 3:**
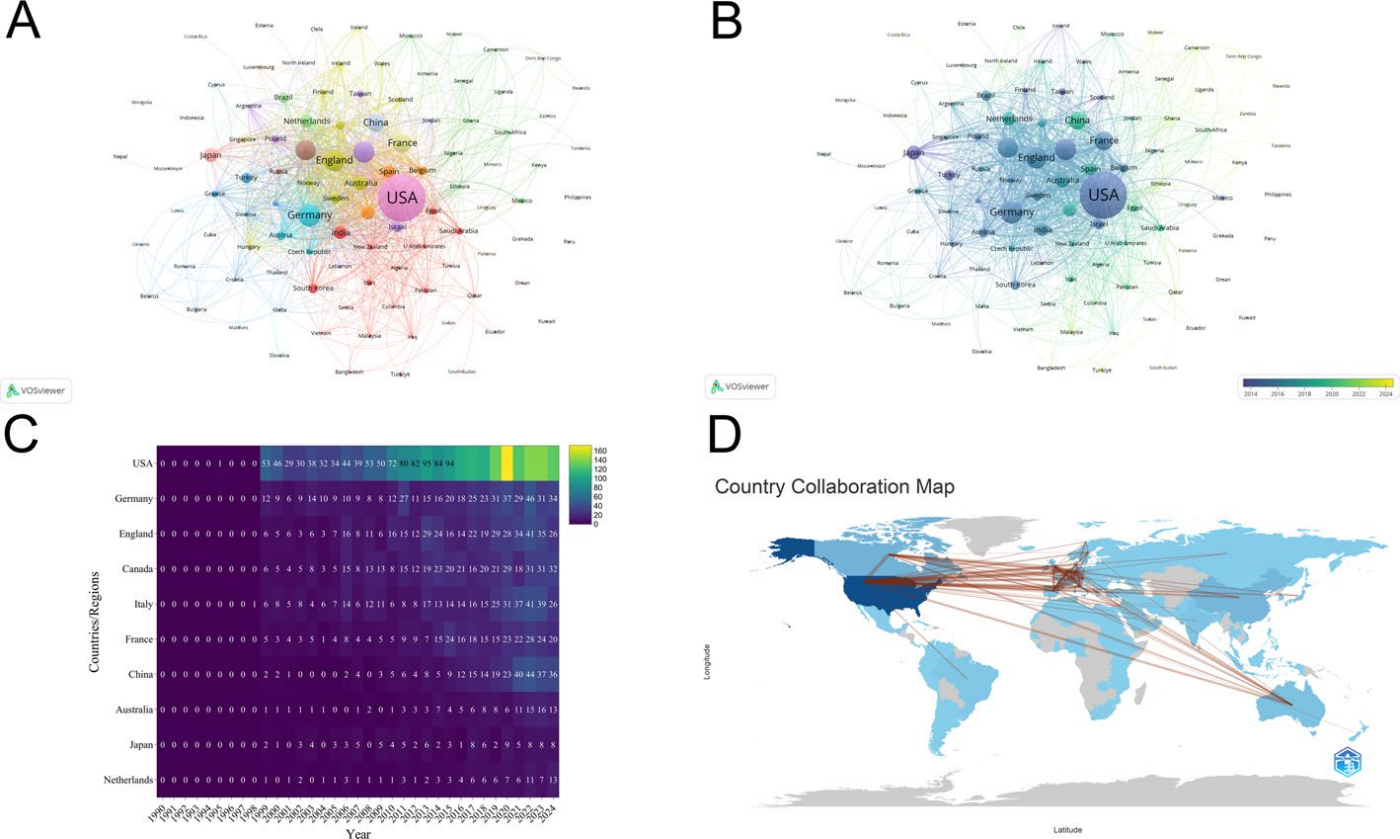
Country cooperation network map. Taiwan is a region of China. **A** Distribution of publications from different countries/regions. The larger the node, the greater the scientific output of that country. The thicker the lines connecting the nodes indicate a deeper cooperation between the countries. **B** Visual mapping of country co-author coverage using VOSviewer. The varying node colors in this visualization reflect the average appearance year (AAY) for each country, represented by the color gradient in the lower right corner. **C** Country/region heat map analysis. The X-axis represents the year, and the Y-axis represents the country/region. **D** The international collaboration among pertinent countries/regions. The closer the color is to dark blue, the greater the output of that country/region. The thicker the line, the closer the cooperation

A total of 4861 documents were published across 109 countries/regions. The top 10 countries/regions with the highest number of publications in the field of PGs are listed in Table 1. The USA published a total of 2118 related papers, accounting for 43.57% of the total, ranking first in terms of quantity, followed by Germany (*n* = 479, 9.85%) and England (*n* = 437, 8.99%). Canada and Italy each contributed 8.25% and 8.19%, with 401 and 398 articles, respectively. These 5 countries accounted for nearly 79% of the total publications. Notably, USA had significantly higher citation counts compared to other countries/regions. We constructed a collaboration network based on publication numbers and relationships among countries/regions, visualizing 109 countries/regions with 1 or more publications (Fig. 3A). The visual analysis shows that the USA is at the core of the network. The width of the lines connecting the nodes indicates that the interaction intensity between the USA and China, England, Germany, and Italy is the highest. In Fig. 3B, according to the average publication year represented by the color of the nodes, the node corresponding to China appears in a lighter shade. This indicates that China entered this research field later than other high-output countries.

According to the heat map data (Fig. 3C), the annual research output time of all countries follows a similar pattern: it remains at a low level (in the single-digit range or zero output) for the first approximately 10 years, and then enters a stage of rapid output growth in the recent 5 years. Throughout the entire period, the annual cumulative research output of the USA is the highest. Among European countries, Germany’s annual output exceeds that of England in the later years. China’s annual output shows the highest growth rate in the later stage, and China’s recent output level has approached that of Germany. Regarding the international cooperation network diagram (Fig. 3D), the research output and cooperation activities in this field are mainly concentrated in economically developed countries in North America, Europe, and East Asia. The USA is at the center of the network and forms the strongest cooperation links with Germany, England, Canada, and Italy. The network as a whole presents a pattern of multiple regional aggregations. Meanwhile, there are fewer direct cooperation links between Australia and the clusters of East Asian countries. These results indicate that the research output in this field shows a general “initial stagnation - later explosion” pattern, which may be related to the evolution law of certain technologies from concept emergence to application maturity. The total output of the USA indicates that it currently plays a leading role in this field. The significant growth in output of Germany and China may indicate the rapid rise of research forces in Europe and East Asia. The “multi-center” structure of the cooperation network indicates that a global research community has formed several regional collaborative alliances; however, compared to other key regional centers, there are significantly fewer cooperation links between Australia and East Asia, and this contrast reveals a major gap in the current cooperation map, which may be a key direction for promoting cross-regional knowledge exchange in the future.

**Table 1:** The top 10 countries with the most publications in the realm of PGs

Rank	Country	Documents	% of (4,861)	Citations	Average citations
1	USA	2118	43.57%	84,720	40
2	Germany	479	9.85%	21,076	44
3	England	437	8.99%	20,539	47
4	Canada	401	8.25%	21,654	54
5	Italy	398	8.19%	9,552	24
6	France	296	6.09%	11,544	39
7	China	291	5.99%	3,783	13
8	Australia	118	2.43%	5,664	48
9	Japan	103	2.12%	2,575	25
10	Netherlands	91	1.87%	3,003	33

### Analysis of affiliations

**Fig. 4:**
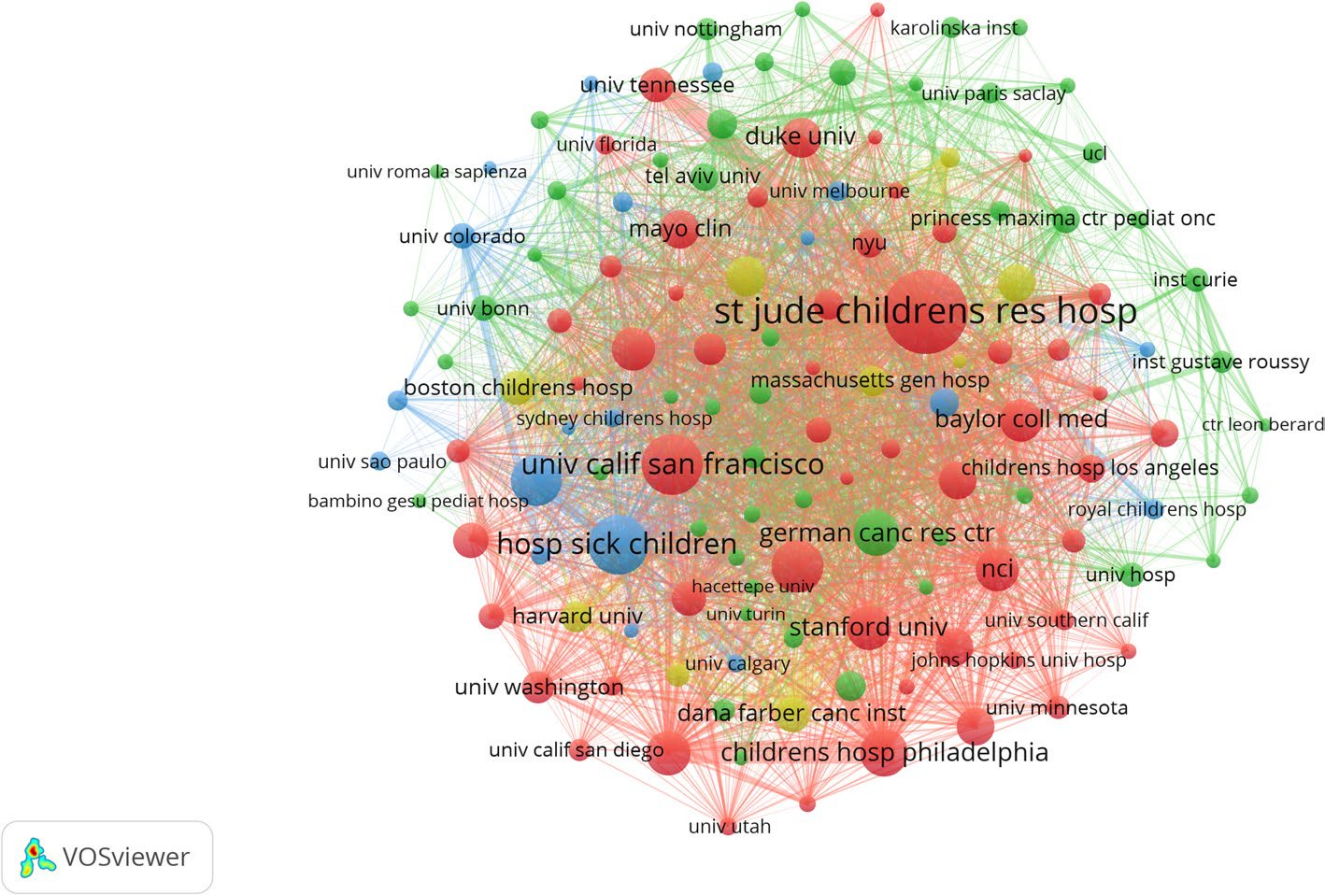
Network diagram of institutions in the field of PGs. The larger the nodes are, the higher the scientific research output of the institution will be. The thicker the lines connecting the nodes, the deeper the cooperation between the institutions will be

St. Jude Children’s Research Hospital ranked first with 294 published papers, followed by the University of California San Francisco (196 papers) and Hospital for Sick Children (175 papers) in second and third place respectively (Table 2). Among the top ten institutions, seven are from the USA. The collaboration network map (Fig. 4, node threshold ≥ 20 papers) visually presents the collaboration relationships among the institutions, with St. Jude Children’s Research Hospital having the largest node and the most numerous and extensive collaboration links. Based on these findings, we can draw the following conclusion: the core position of St. Jude Children’s Research Hospital in terms of output volume and the collaboration network indicates that it is the most influential research hub in the field of pediatric genetics. American institutions occupy the vast majority of seats among high-quality output institutions, which confirms the leadership of the USA in this research field at the institutional level. The form of the collaboration network reveals that knowledge flow may highly depend on a few core institutions.

**Table 2:** The top 10 institutions in terms of the number of publications in PGs

Rank	Institution	Country	Publications	Citations	Average citations
1	St. Jude Children’s Research Hospital	USA	294	5,830	20
2	University of California San Francisco	USA	196	7,186	37
3	Hospital for Sick Children	Canada	175	2,744	16
4	University of Washington	USA	151	5,577	37
5	University of Toronto	Canada	134	3,296	25
6	Children’s Hospital of Philadelphia	USA	128	3,878	30
7	Children’s National Medical Center	USA	119	973	8
8	German Cancer Research Center	Germany	114	4,787	42
9	Baylor College of Medicine	USA	112	8,353	75
10	Stanford University	USA	110	4,432	40

### Analysis of authors

**Fig. 5:**
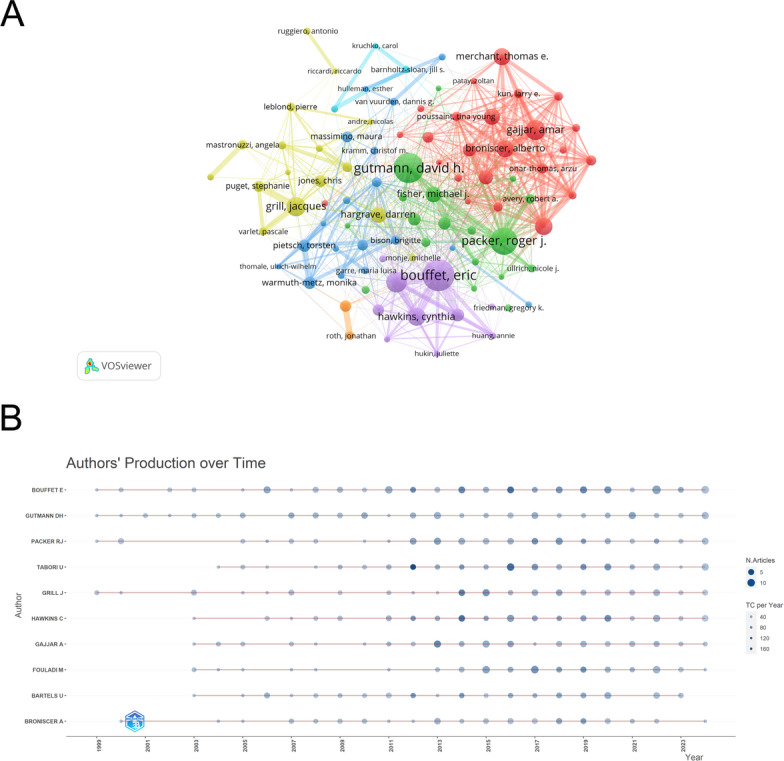
Authors related to the research on PGs. **A** The visualization map of authors. The larger the node, the greater the amount of scientific research output. The thicker the lines connecting the nodes, the more active the corresponding cooperative connections are. **B** The top 10 authors with the highest volume of article production from 1961 to 2024. The X-axis represents the years, and the Y-axis shows the top 10 authors with the highest productivity in the PGs field. The darker the node color is, the more citations it has received

The authors’ contributions were assessed according to their publication output [52]. A total of 23,161 authors participated in the research in this field. Figure 5A, based on the author visualization analysis using VOSviewer (with a threshold of ≥ 15), generated a network graph containing multiple clusters, identifying a total of 7 main clusters. Table 3 lists the top 10 most productive authors in the PGs field. Additionally, Fig. 5B shows the top 10 authors who published the most papers from 1961 to 2024; in this Fig. 5B, the size of the circles is proportional to the academic output, and the color intensity is proportional to the number of citations. Based on these findings, we make the following interpretation: the different clusters in Fig. 5A may represent different research directions or academic groups. Among them, the obvious close connections within the red, green, and purple clusters suggest that there are stable and active cooperative relationships within these groups. Authors such as Bouffet Eric and Gutmann Dh had earlier academic outputs, indicating that they are pioneers in this field, and their work laid the foundation for subsequent research. And some authors who began to publish results after 2000 also rank high on the list of productive authors, indicating that this field is continuously attracting new research forces and maintaining continuous academic vitality. In summary, these high-output and highly-cited authors jointly shape and drive the development of this discipline.

**Table 3:** The top 10 authors with the highest number of publications in PGs

Rank	Authors	Country	Articles	Citations	Average citations
1	Bouffet E	Pakistan	122	3017	24.73
2	Gutmann Dh	USA	94	1553	16.52
3	Packer Rj	USA	87	1920	22.07
4	Tabori U	Canada	75	1983	26.44
5	Grill J	French	74	1508	20.38
6	Hawkins C	Canada	73	2047	28.04
7	Gajjar A	USA	69	1135	16.45
8	Fouladi M	USA	66	1290	19.55
9	Bartels U	Canada	55	1752	31.85
10	Broniscer A	USA	55	822	14.95

### Analysis of journals

**Fig. 6:**
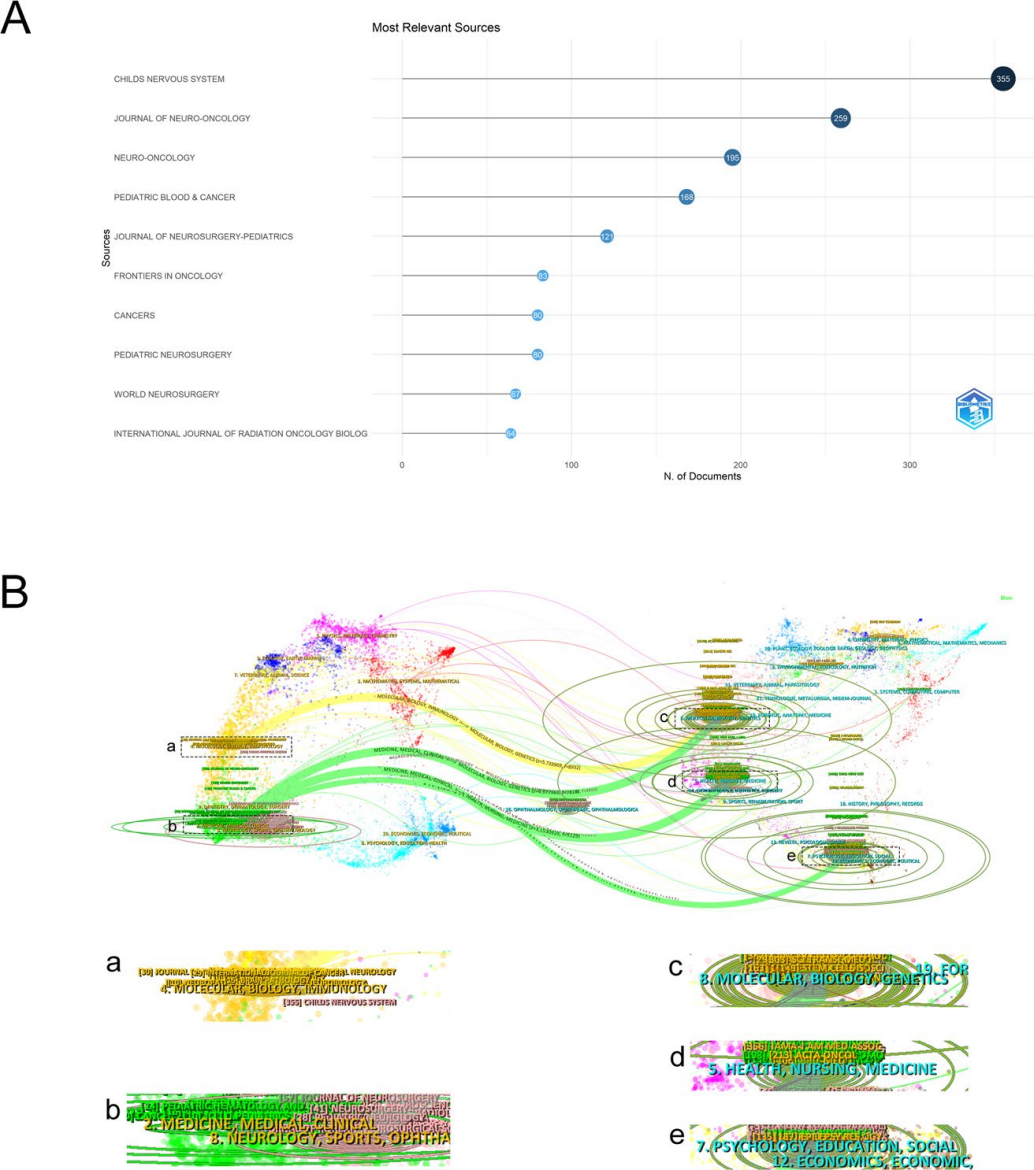
Analysis of relevant journals in the PGs field. **A** The scientific publication sources of the top 10 journals and the number of articles related to PGs published. **B** Journal double-graph overlay analysis. The left and right parts of the graph respectively show the thematic coverage of the citing and cited references. The citation relationships in different academic fields are represented by connecting lines, and the thickness of the lines reflects the strength of the relationship

Research on PGs has been disseminated across 956 academic journals. The top 10 journals with the most publications in this field are depicted in Fig. 6A. Childs Nervous System leads, having published 355 articles. Table 4 enumerates these leading journals, including their citation metrics and impact factors. The citation count (7,900), H-index (54) and impact factor (16.4) of Neuro-Oncology are all the highest among all high-productivity journals. Based on the above data, it can be inferred that although the publication volume of Childs Nervous System is the largest, Neuro-Oncology holds the leading position in terms of citation influence. This might indicate that the research published by Neuro-Oncology has had a more extensive academic impact within the PGs field.

The journal double-map overlay analysis in CiteSpace visualizes the thematic distribution and interdisciplinary relationships among scientific domains [53]. In Fig. 6B, the left and right panels represent the thematic coverage of the citing references and cited literature, respectively. Citation linkages between domains are denoted by connecting lines, with line thickness indicating the strength of association. Notably, the domain “MOLECULAR, BIOLOGY, IMMUNOLOGY” draws upon foundational knowledge from “MOIECULAR, BHOLOGY, GENETICS”. Similarly, “MEDICINE, MEDICAL, CLINICAI” is primarily underpinned by contributions from “HEALTH, NURSING, MEDICINE”, “MOIECULAR, BHOLOGY, GENETICS” and “PSYCHOLOGY, EDUGATION, SOCIAL”.

**Table 4:** The top 10 journals in terms of the number of publications relating to PGs

Rank	Source	Documents	% of (4861)	Citations	IF	H-index
1	Childs Nervous System	355	7.30%	4445	1.3	34
2	Journal of Neuro-Oncology	259	5.33%	6345	3.2	44
3	Neuro-Oncology	195	4.01%	7900	16.4	54
4	Pediatric Blood & Cancer	168	3.46%	3353	2.4	38
5	Journal Of Neurosurgery-Pediatrics	121	2.49%	1525	2.1	27
6	Frontiers In Oncology	83	1.71%	1126	3.5	19
7	Cancers	80	1.65%	1347	4.5	30
8	Pediatric Neurosurgery	80	1.65%	33	0.9	23
9	World Neurosurgery	67	1.38%	1	1.9	15
10	International Journal of Radiation Oncology Biology Physics	64	1.32%	3	6.4	36

### Analysis of co-cited references

**Fig. 7:**
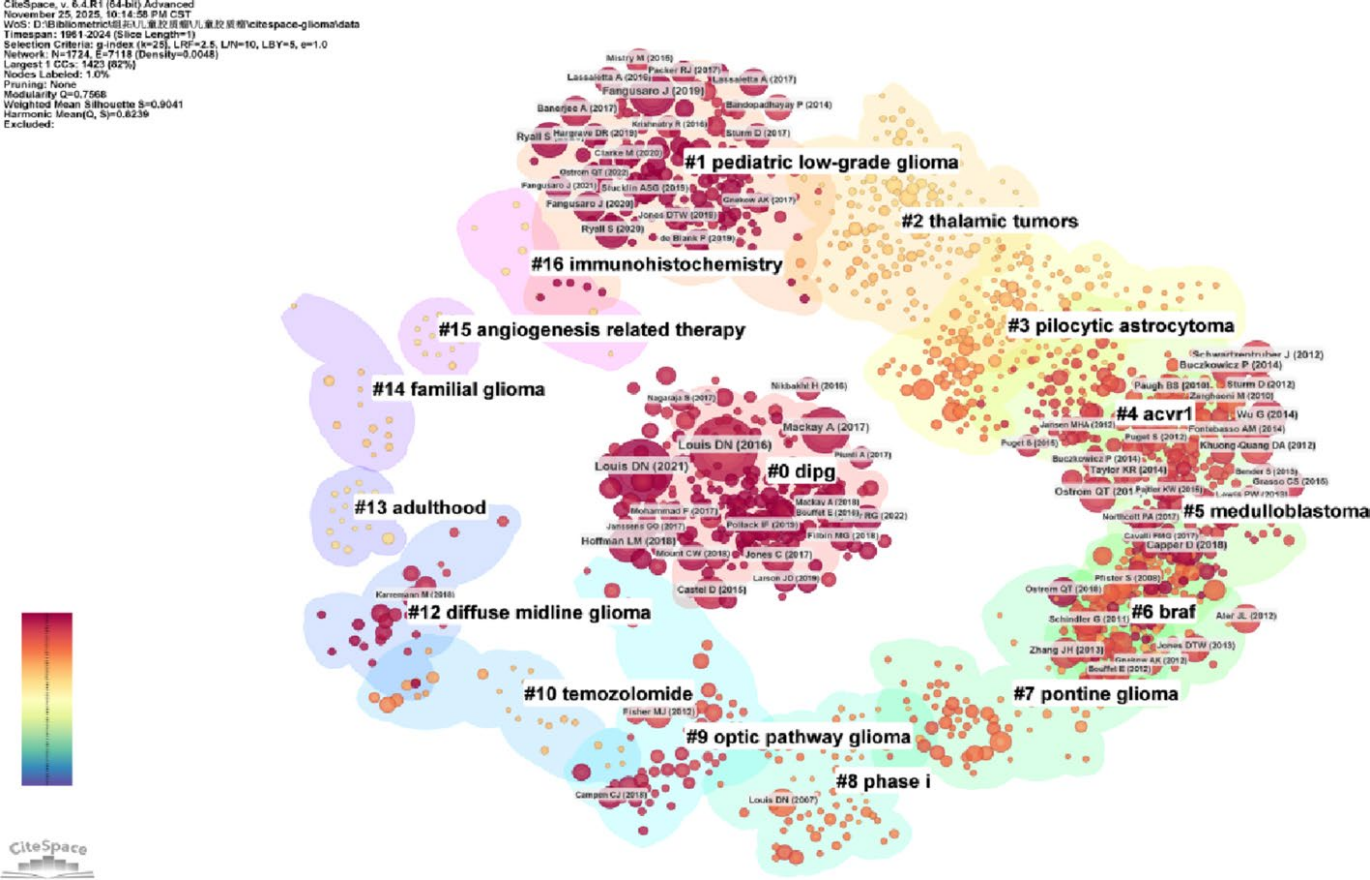
The cluster analysis of highly co-cited references in the field of PGs. Node color represents the average publication year; colors closer to red indicate nodes appearing more recently. The larger the number of nodes within the same cluster, the greater the influence of the keywords in that cluster. The larger the node in the cluster, the more times the paper has been cited

According to reports, two papers jointly cited by another document exhibit a co-citation relationship [52]. We constructed a comprehensive co-citation network using CiteSpace to identify pivotal publications, knowledge bases, and emerging research frontiers in the field. Figure 7 presents the synthesized reference co-occurrence map, covering publications from 1961 to 2024, where node selection prioritized citation frequency and centrality. The analysis results show a total of 16 research clusters. Among them, the “pediatric low-grade glioma” (cluster #1) and “dipg (diffuse intrinsic pontine glioma)” (cluster #0) related to PGs have become the most intensively studied fields in recent years.

### Analysis of keyword co-occurrence

**Fig. 8:**
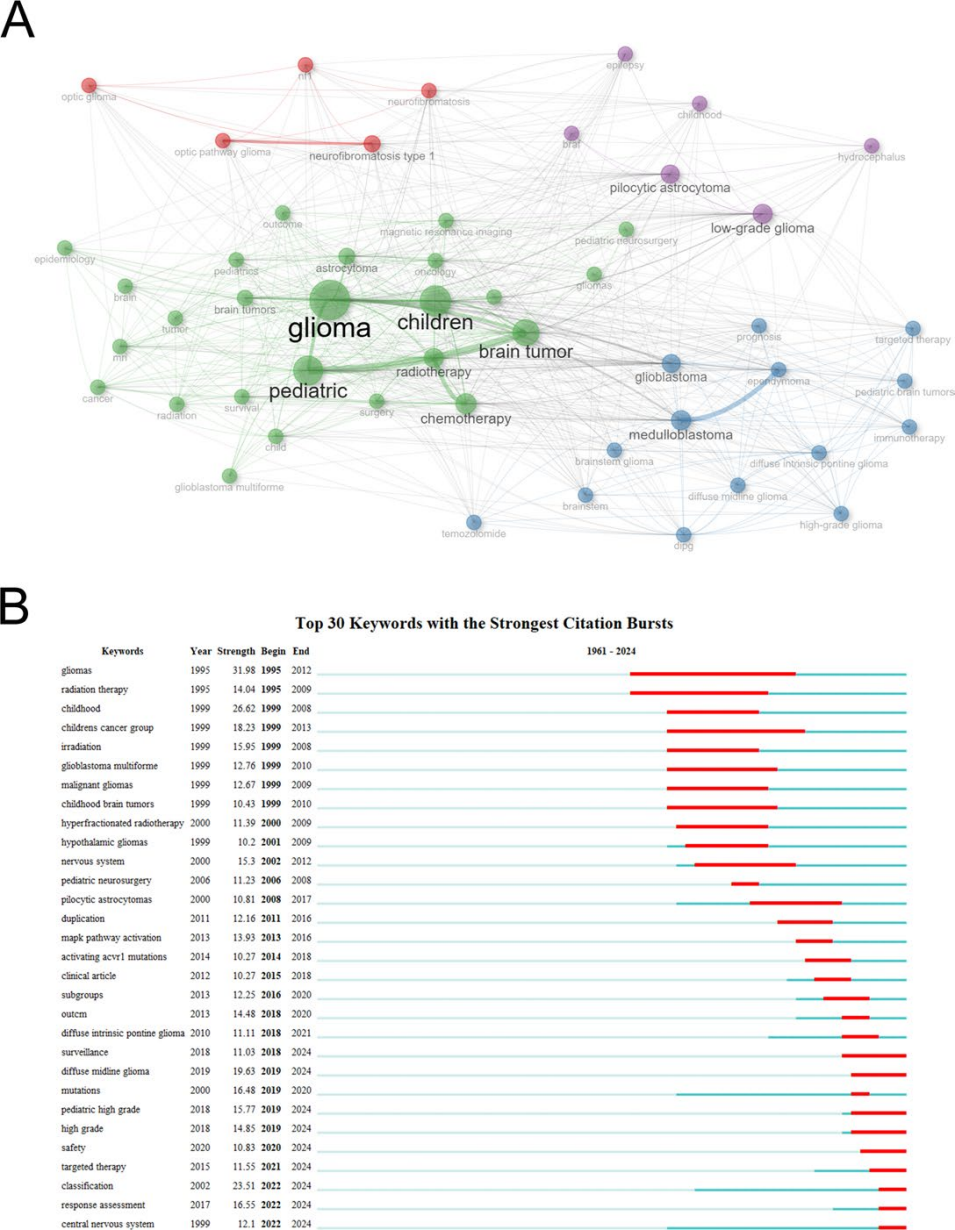
Visualized analysis of keywords and literature related to PGs. **A** The terms co-occurrence network of the 4,861 documents. Nodes represent keywords (Top 50). Lines refer to the co-occurring relationship. **B** Top 30 keywords with the strongest citation bursts. Burst strength and time duration of the top 30 keywords with the strongest citation bursts, where blue lines represent the research time dimension, and the highlighted red segments indicate significant research frontier mutation periods. Each mutation period is precisely labeled with the start year, end year, and duration

Keyword co-occurrence analysis is an effective bibliometric method for capturing current themes in scientific knowledge structure research [54]. Based on the frequency of keyword occurrence, the keyword co-occurrence network (Fig. 8A) generated four main clusters, each labeled in different colors: the core keywords of the green cluster include “glioma” and “children”. The core keywords of the blue cluster include “medulloblastoma”, “high-grade glioma”, “targeted therapy”, “immunotherapy”. The core keywords of the purple cluster include “low-grade glioma”. The core keywords of the red cluster include “neurofibromatosis type 1”. The keyword emergence analysis (Fig. 8B) revealed the changes in the influence of different keywords over different periods: the most intense emergent words in the early period (1995–2010) included “gliomas” (intensity: 31.98, 1995–2012) and “glioblastoma multiforme” (intensity: 12.76, 1999–2010). The emergent words in the middle period (2010–2020) included “MAPK pathway activation” (intensity: 13.93, 2013–2016), “activating ACVR1 mutations” (intensity: 10.27, 2014–2018), “subgroups” (intensity: 12,25, 2016–2020), and “diffuse intrinsic pontine glioma” (intensity: 11.11, 2018–2021). The emergent words with significant intensity in the recent period (2018–2024) included “diffuse midline glioma” (intensity: 19.63, 2019–2024), “pediatric high grade” (intensity: 15.77, 2019–2024), “targeted therapy” (intensity: 11.55, 2021–2024), and “response assessment” (intensity: 16.55, 2022–2024). Based on the clustering structure of the co-occurrence network, the research in pediatric neuro-oncology has formed several clear directions: the green cluster focuses on the clinical diagnosis and traditional treatment of PGs; the blue cluster focuses on molecular targeting and immunotherapy for specific tumor types; the purple and red clusters correspond to the two subfields of LGGs and neurofibromatosis associated with syndromes. The time series of emergent words clearly outlines the paradigm shift in research. The highly intense emergent words in the early period were related to traditional treatment methods (such as radiotherapy) and basic disease classification, reflecting the priority of meeting urgent clinical needs at that time. The emergent words in the middle period shifted to “MAPK pathway”, “molecular mechanisms”, and “molecular typing”, marking the deepening of research, from traditional treatment to the era of precise classification and targeted therapy for diseases. The emergent words in the recent period, such as “diffuse midline glioma” and “targeted therapy”, indicate the focus on refractory tumors and the deepening application of precision medical technologies as current hotspots. At the same time, the emergence of “response assessment” highlights the increasing emphasis on the scientific quantification of treatment outcomes in the context of new therapies.

### Analysis of hotspots and trends in research

**Fig. 9:**
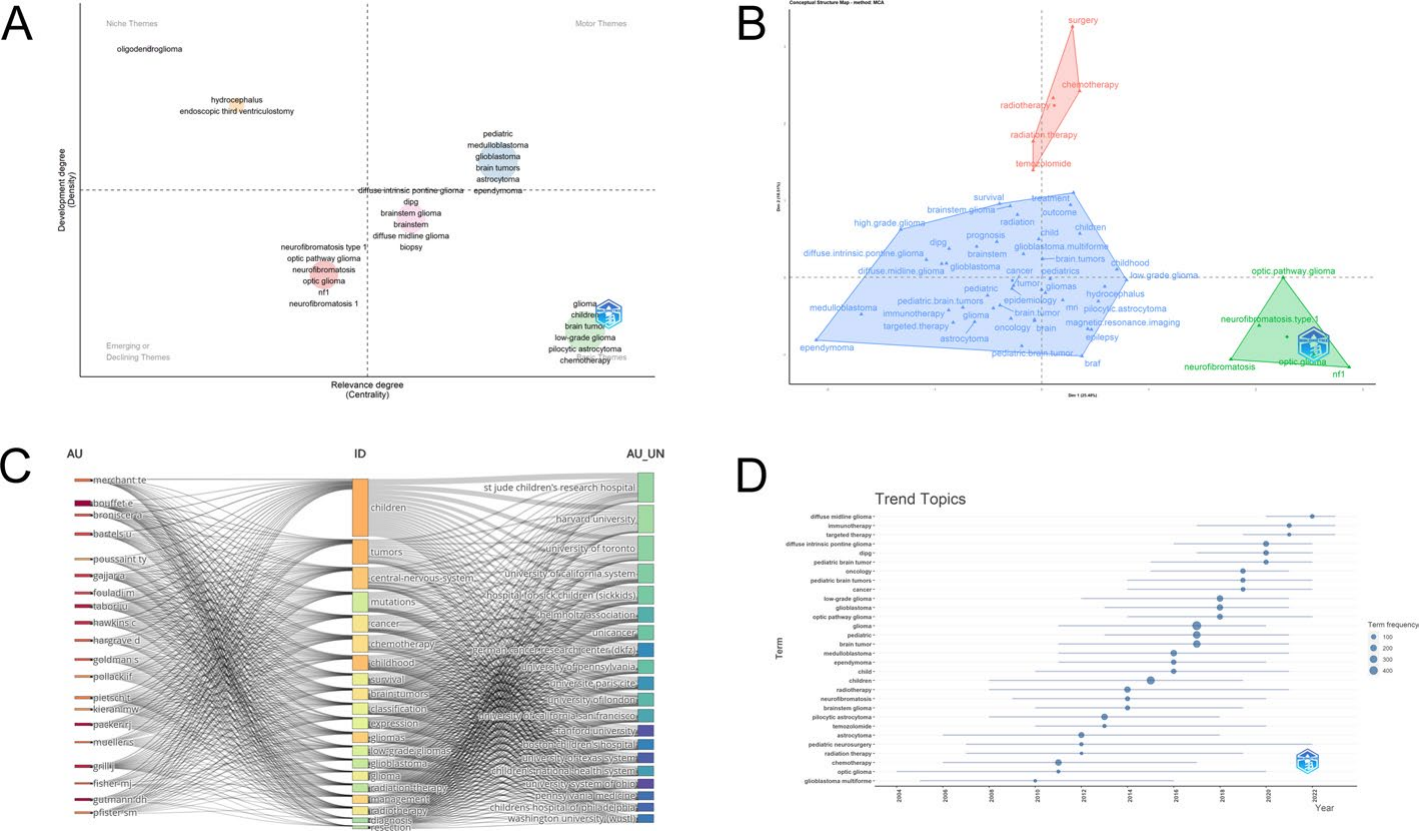
Analysis of thematic words and keywords. **A** Thematic analysis in the field of PGs. The horizontal and vertical axes represent centrality and density, respectively. The first quadrant is well-developed topics, the second quadrant is not important to the current field, the third quadrant is topics that may have recently emerged or may soon disappear, and the fourth quadrant is basic topics that are not important. **B** Conceptual structure map of Keyword Plus. **C** The association and network diagram of academic terms and research institutions. Academic terms (in the middle column “ID”) are associated with research institutions (in the right column “AU_UN”) and authors (in the left column “AU”). The width of the arcs corresponds to the strength of the association (the closer the association, the wider the arc). **D** Timeline of research trends in the field of PGs. The X-axis represents the year, and the Y-axis represents the keyword. The size of the nodes represents the frequency of occurrence of the key terms

This study employed thematic analysis to explore the core issues in the PGs field. Figure 9A identified several keyword sets, including those centered on “pediatric”, “medulloblastoma”, “glioblastoma”, “brain tumors”, “astrocytoma”, and “ependymoma”; those centered on “neurofibromatosis type 1”, “optic pathway glioma”, “neurofibromatosis”, “optic glioma”, “nf1”, and “neurofibromatosis 1”; And the third vocabulary collection includes words such as “dipg” and “diffuse midline glioma”; the fourth one contains key words like “low-grade glioma” and “chemotherapy”. Among these sets, the first one represents the core of basic and mature research in this field; the second one indicates that subtype analysis based on specific diseases is a continuous focus; and the third one highlights some important subtypes; the fourth one focuses on the basic classification of PGs and the basic knowledge of clinical treatment. The distribution pattern of “chemotherapy” in the Fig. 9A further supports the evolution of the research focus from traditional chemotherapy to new strategies such as targeted and immunotherapy.

The high-frequency keywords were classified using the multi-dimensional scaling method. We constructed a conceptual structure diagram (Fig. 9B) consisting of three clusters (red, blue, and green). The red cluster includes “surgery”, “chemotherapy”, “radiotherapy”, “radiation therapy”, and “temozolomide”. The blue cluster contains keywords such as “MRI”, “BRAF”, “survival”, “brainstem glioma”, “dipg”, and “immunotherapy” and “targeted therapy”. The green cluster includes “nf1”, “optic.pathway.glioma”, “neurofibromatosis. type.1”, “neurofibromatosis”, etc. Based on the above clustering results, we speculate that the keyword combination in the red cluster indicates that this cluster mainly represents the basic and traditional treatment methods. The blue cluster links diagnostic techniques (magnetic resonance imaging), molecular markers (BRAF), specific diseases (brainstem glioma, medulloblastoma), and clinical outcomes (survival rate), highlighting the close integration of diagnosis and prognosis assessment in modern neuro-oncology and emphasizing the core role of molecular markers and imaging in the precision medical paradigm. “Immune therapy” and “targeted therapy” may also reflect their status as emerging treatment paradigms in development. Common pediatric conditions such as “neurofibromatosis type 1”, “optic pathway glioma”, “neurofibromatosis”, and “optic glioma” are also prevalent in the research.

The R-bibliometrix tool was used to draw a three-dimensional overlay chart of keywords, authors and institutions to analyze the correlations among them (Fig. 9C). The chart shows that the frequently used keywords “mutations”, “chemotherapy”, “survival” and “brain tumors” are spatially close to each other. The main research institutions shown in the chart include St. Jude Children’s Research Hospital, the University of Toronto, the University of California system, Harvard University, etc. Moreover, most of the high-productivity institutions are located in North America. Our interpretation of these findings is as follows: the close association of the keywords “mutation”, “chemotherapy” and “survival” indicates that one of the current research focuses is to explore the intrinsic connection between tumor gene mutations and chemotherapy responses as well as patient prognosis. This may involve in-depth exploration of the mechanisms of chemotherapy resistance. The phenomenon that the majority of the high-productivity institutions are dominated by North American institutions reflects to a certain extent that this region holds a dominant position in pediatric neuro-oncology research. This might be due to the more concentrated scientific research resources in this region.

The temporal trend of the keyword occurrence frequency has been visualized (Fig. 9D). This graph shows that the occurrence frequencies of the keywords “immunotherapy” and “targeted therapy” have shown a significant upward trend in the recent period (e.g., 2018–2024). In contrast, the frequencies of the keywords “chemotherapy” and “radiation therapy” remained stable or slightly decreased during the same period. Meanwhile, the frequency of the keyword “diffuse midline glioma” has shown a significant initial increase in the recent period. Based on the above temporal trend, we believe that the increase in the frequency of “immunotherapy” and “targeted therapy” indicates that they have become the current most focused emerging research directions in this field. While the stable trend of the frequencies of “chemotherapy” and “radiation therapy” suggests that their status as basic treatment methods remains stable, they may no longer be the main growth points of academic innovation. Considering the clinical challenges in this field, we believe that the rapid increase in the attention to “diffuse midline glioma” precisely reflects that the academic community is concentrating its efforts on addressing this disease with extremely poor prognosis and limited treatment options, making it an emerging key research target.

## Discussion

### Interpretation of principal findings

This bibliometric analysis comprehensively maps the landscape of PGs research, examining countries, journals, citations, collaborations, and author contributions over time. From 1961 to December 2024, the research topics in this field have shown a significant expansion trend. The research results indicate that academic achievements related to PGs have been increasing. The top few journals ranked by H-index are Neuro-Oncology, Journal of Neuro-Oncology, and Pediatric Blood & Cancer. St. Jude Children’s Research Hospital is the research institution that has produced the most. The USA leads in PGs research, followed by Germany, England, and Canada, which also have the largest number of active research institutions in these countries. Over time, the research topics in the PGs field have gradually shifted from traditional treatment methods such as surgery, chemotherapy, and radiotherapy to targeted therapy, immunotherapy, and molecular classification. This indicates that the medical field is moving towards the path of precision medicine and integrated multi-omics approaches [49, 51]. The 2016 WHO update to CNS tumor classification, incorporating molecular markers like IDH and H3K27M mutations, marked a pivotal moment [55]. This reclassification had a particularly significant impact on diffuse midline gliomas with the H3K27M mutation. Subsequently, the research focus shifted to targeted intervention and molecular pathological mechanisms [56].

### International collaboration and research

Data analysis shows that the PGs research field has established a relatively stable international cooperation framework, and the USA has become the center of global research activities. Not only does the USA rank first in the number of published papers, but it has also established extensive cooperation networks with countries such as Germany, England, and Italy, forming multi-center research alliances. This collaborative research situation is very important for advancing complex PGs research, especially in the fields of molecular research and clinical trials that require a large amount of resources. The professional knowledge and research conditions of each country work together, resulting in very obvious effects. It is worth noting that China’s role in this network is becoming increasingly prominent, which indicates that the global research landscape is changing. This also shows that Asian institutions are making more and more contributions in the field of pediatric neuro-oncology. Germany’s dominant position may be related to the relatively mature development of its pediatric oncology projects. Europe mainly focuses on translational neuro-oncology research. Canada’s significant contribution has strengthened North America’s leading position in PGs research. This distribution pattern also enables us to more clearly see the differences in the positioning of different regions in the research landscape. The cooperation among high-income countries is very close, which shows that the availability of resources has a significant impact on the research output and innovation speed in this field. Previously, the progress in PGs treatment was initially based on histopathological classification and traditional therapies. However, due to the lack of molecular typing, these treatment methods often encountered many difficulties [57]. In the early 21st century, the emergence of high-throughput sequencing technology promoted the development of precise molecular typing [58]. Especially in the 2021 WHO revision, the H3K27M mutant glioma was classified as an independent and highly invasive subtype [59, 60], which accelerated the pace of targeted therapy research. In recent years’ research (from 2022 to present), there is a notable feature, which is the integration of multi-omics information and the use of artificial intelligence (AI) for diagnosis. Projects such as the Children’s Brain Tumor Consortium (CBTTC) and Children’s Brain Tumor Network (CBTN) are typical examples. These projects bring together the forces of global institutions and rely on data sharing to enhance the level of personalized treatment [61]. Breakthroughs in genomics, epigenetics, and immunology have propelled targeted therapies (e.g., brigatinib, everolimus) and immunotherapies (e.g., CAR-T [chimeric antigen receptor-T]) to prominence, substantially improving patient survival. In the precise treatment of children with glioma, there is a notable advancement worth noting. Jiang Tao’s team, relying on the Chinese glioma gene map, extended the median survival period of patients from 17.5 months to 29.3 months [62, 63]. Additionally, international clinical trials have also demonstrated that everolimus is effective for recurrent or progressive LGGs, providing more solid evidence for stratified treatment [63].

### Journal studies

The bibliometric analysis shows that there are distinct divisional characteristics among the journals in the field of PGs research. Childs Nervous System published 355 papers, accounting for 7.30% of the total output, ranking first in terms of quantity. This indicates that it plays a significant role in integrating clinical case resources and conducting regional observational studies. However, the impact factor of this journal is not high (1.3), and its H-index is only 34. This might be related to its publication characteristics - it has always focused on case reports and clinical observational studies. Although such studies are important for accumulating knowledge, they generally have a relatively narrow citation range, with each paper being cited approximately 12.5 times on average. Neuro-oncology mainly focuses on conducting mechanism studies and validating innovative therapies [64, 65]. It is worth noting that adult oncology-related journals are relatively marginalized in the pediatric field, which further highlights the uniqueness of this subfield [66, 67]. For instance, the highly influential and prestigious journal International Journal of Radiation Oncology Biology Physics (with an IF of 6.4 and an H-index of 36) in adult radiation oncology has significantly less influence in the field of PGs research. This journal has published 64 papers related to PGs, but the total number of citations is only 3, and the average citation per paper is less than 0.05. This clearly shows that PGs and adult gliomas have fundamental differences in pathophysiology, treatment tolerance, and long-term side effects. Therefore, it is highly necessary to establish specialized research methods and publishing platforms. The PGs research field is defined by a large number of influential journals. In sharp contrast to this is Neuro-Oncology (with an IF of 16.4 and an H-index of 54). Although the number of papers it publishes is only 55% of that of Childs Nervous System (195 papers, accounting for 4.01% of the total output), its total citation count (7,900 times) and the average citation count per paper (approximately 40.5 times) are much higher than the latter, indicating that it holds a dominant position in driving the paradigm shift progress in this field. The advantage of this influence might be due to its consistent focus on research that can bring about significant changes. This journal was the first to publish important findings related to brainstem gliomas, such as in the molecular mechanisms (like the characteristics of H3K27M mutations in DIPGs) and in targeted therapies (such as the application of BRAF inhibitors in LGGs) [68–70]. Such studies, due to their profound theoretical basis and potential for clinical application, have attracted many people to continue their research (highly cited), and may even rewrite treatment guidelines.

The PGs research has gradually formed a research community, with two core areas playing a leading role: one is the Childs Nervous System, and the other is Neuro-Oncology. The Childs Nervous System functions like a platform for integrating clinical resources, as it has published the most papers and possesses a large amount of case observation data, laying the foundation for researchers to comprehensively understand various PG diseases. Neuro-Oncology, on the other hand, is like an engine for cutting-edge innovation, relying on influential new discoveries to continuously deepen the research on molecular classification and targeted therapy. These two journals collaborate together, accumulating large-scale clinical data on the one hand, and conducting influential research projects on the other, making the entire research circle both complete and full of vitality for progress. This also makes these two journals the structural cornerstone for the advancement of PGs research.

### Development of research themes

The research on PGs has shifted from descriptive pathology in the past to a new direction of precision medicine. In the initial stage (from 1995 to 2010), the research mainly focused on conventional therapies (such as radiotherapy and chemotherapy) and tissue morphology classification [71]. This can be seen from the frequent keywords like “radiotherapy” and “malignant glioma”. The research also found that classification based on tissue morphology could help predict the disease progression - for children with glioblastoma (GMB), the 4-year overall survival rate was only 30%, which was significantly lower than that of patients with anaplastic astrocytoma or oligodendroglioma (58%) [72]. Maura et al. proposed a dense sequential chemotherapy method in 2005. First, use cisplatin and etoposide for guiding treatment, then add vincristine and cyclophosphamide, and add high-dose methotrexate. They also innovatively placed high-dose teniposide (300 mg/m², taken in three doses) before radiotherapy as a “sandwich strategy” to integrate with radiotherapy [72–74]. To support this regimen, autologous stem cell transplantation was employed for myeloprotection during high-dose chemotherapy [74, 75]. However, even with these advancements, the 4-year overall survival rate was only 43%, and the progression-free survival (PFS) rate was only 46%, which was still not good enough [71]. Nevertheless, these early explorations were not in vain; they not only established the benchmark for treatment effectiveness but also paved the way for subsequent optimization of the plan. This step-by-step research gradually found a more feasible direction.

After 2010, the research direction of PGs shifted to targeting specific targets. At that time, researchers discovered the activation of the MAPK pathway and ACVR1 mutations in PGs. Whole-genome studies indicated that changes in the MAPK pathway (such as BRAF fusion, newly emerging FGFR1/NTRK2 fusion, and KRAS/NF1 mutations) could be found in 100% of pilocytic astrocytomas (PAs) [76], confirming its characteristic as a “single-pathway disease” [77–79]. Additionally, in 21% of DIPGs, somatic ACVR1 activation mutations (such as R206H, G328V) were repeatedly observed, which were similar to the genetic mutations in fibrous ossification progressive syndrome (FOP). These mutations would continuously activate the BMP/TGF-β pathway and make the tumors responsive to ALK2 inhibitors [77, 80–82]. FGFR1 mutations were also found in non-cerebellar PAs, and in the subgroup of children with GMB carrying H3F3A K27M and NF1 mutations, FGFR1 mutations were also discovered [83, 84]. It was also noted that BRAF inhibitors might cause “abnormal activation” in fusion-driven tumors [85]. These factors led to the initiation of research on the combination of specific inhibitors targeting FGFR, NTRK2, MEK, and BRAF V600E [85–87]. These findings clarified the link between ACVR1 gene mutations and specific subtypes of DIPG, which can slightly prolong the survival period of patients [88]. They also laid the foundation for precise targeted therapy based on molecular subtypes. After the 2021 update of the WHO classification, molecular markers (such as H3K27M and BRAF) were officially included in the diagnostic criteria [89–92], further accelerating this transformation process.

The recent research (from 2018 to 2024) mainly focused on new disease types (such as DMG), technological advancements (such as AI-assisted diagnosis), and immunotherapy, indicating that the research field of PGs is moving towards personalized treatment strategies. The research also identified mutations coexisting with the H3K27M mutation, such as ACVR1, TP53 [93, 94], which has driven the development of personalized targeted therapy. For instance, the GD2-CAR-T therapy has shown clinical effects in patients with H3K27M mutations [95]. AI and radiomics have been applied to noninvasive diagnostics, such as MRI-based radiomic models predicting H3K27M status (AUC = 0.97) [96] and survival prognosis (C-index = 0.81) [97]. Treatment is no longer solely dependent on checkpoint inhibitors, but has shifted to a multimodal strategy. For instance, the combination of H3K27M-targeted peptide vaccine with nivolumab has significantly prolonged the survival period of children with glioma [98]. Combining focused ultrasound (FUS) with chemotherapy can enhance the permeability of the blood-brain barrier; while in recurrent cases, combining radiotherapy with re-irradiation can also improve survival rates. These advancements indicate that the treatment approach is shifting from the traditional combination of chemotherapy and radiotherapy to molecular-guided personalized multimodal therapy [99–101].

### Dynamics of keywords

The co-occurrence and emergence analysis of key words indicates that the research hotspots have distinct temporal characteristics. Words like “chemotherapy” and “radiotherapy”, along with emerging keywords such as “targeted therapy” and “immunotherapy”, appearing together, suggest that we are currently in a transitional period where traditional treatment methods and precision medical approaches are cooperating with each other. Immunotherapy works by activating T cells to directly eliminate tumor cells [102], while targeted drugs achieve this by inhibiting signaling pathways, thereby indirectly controlling the growth of tumors [40]. Over 90% of pLGGs exhibit RAS/MAPK pathway activation, which serves as the molecular foundation for targeted therapies [103]. The underlying mechanisms vary significantly by driver alteration. The following examples illustrate the different targeted therapies that are suitable for different mutations. BRAF V600E missense mutations drive tumorigenesis through constitutive kinase domain activation [103, 104]. Targeted drugs like dabrafenib can directly bind to the ATP binding site of the mutant protein, thereby blocking abnormal signal transduction [85, 105]. Some BRAF fusion genes, such as KIAA1549-BRAF, exhibit kinase activity that is not controlled by ligands. First-generation BRAF inhibitors can instead activate wild-type RAF dimers, so researchers have developed second-generation drugs like PLX8394 that can inhibit dimers and thus cut off their signal transduction [85]. NF1 loss-of-function mutations inhibit RAS GTPase activity, so MEK inhibitors like selumetinib are needed to suppress the phosphorylation of downstream ERK [24]. If FGFR1 is altered, such as by tyrosine kinase domain duplication, the receptor kinase will be continuously activated. FGFR-specific inhibitors like AZD4547 can block the phosphorylation of the receptor and thereby inhibit both the MAPK and PI3K pathways [76]. There are also some rare fusion kinases, such as NTRK or ALK fusions, which induce tumorigenesis through the dimerization of fusion proteins. These proteins can be effectively targeted by ATP-competitive inhibitors like larotrectinib [106]. In pHGGs, many immune-targeted strategies have been developed. CAR-T is one of them, which can recognize surface antigens like GD2/B7-H3 and directly act on tumor cells [102, 107]. When it comes to CAR-T therapy, Dash et al. proposed a new approach. Their new therapy is based on CRISPR-based gene editing therapy (based on the HIV model) and has been verified in animal experiments, being capable of completely eliminating diseases in the mammalian system. This verification provides support for the application of other gene editing therapies such as CAR-T-related treatment methods [108]. Second, combination strategies demonstrate enhanced efficacy: concurrent oncolytic viruses (e.g., IL-7-armed adenoviruses) boost T-cell infiltration [109], while immune checkpoint inhibitors (e.g., anti-PD-1 antibodies) improve T-cell persistence [110]. The hotspots and trends in future PGs treatments will focus on targeted immunotherapy, as well as precise and personalized therapies.

The continuous emphasis on the keywords “mutation” and “survival” highlights the ongoing necessity of linking genomic discoveries with clinical outcomes. Ryall et al. conducted a comprehensive genomic analysis of over 1,000 cases of pLGGs, and the results revealed that 84% of the cases had clear driver mutations. This was the first time that the role of mutation types (rearrangement-driven and point mutation-driven) as key prognostic factors was clarified. At the same time, it emphasized that when conducting molecular subtype analysis, the mutation mechanism (fusion/SNVs) must be considered to optimize clinical risk stratification [5]. This classification directly informs targeted strategy selection: BRAF V600E point mutations respond to type I RAF inhibitors, whereas BRAF fusions require type II RAF inhibitors (e.g., tovorafenib) to avoid paradoxical activation [111–113]. Some high-risk mutation combinations (such as BRAF p.V600E combined with CDKN2A deletion, or H3F3A p.K27M) define subgroups with extremely poor prognosis: the 10-year PFS rate is 0%, and the overall survival rate is only 35% to 60%. These high-risk subgroups require early use of combined targeted and other intensified treatments. While the low-risk group (such as tumors with fusion genes or NF1 mutations) does not need excessive treatment. This shows that analyzing the mutation profile is of great clinical value in personalized management [5, 114]. In pHGGs, tumors with TP53 mutations respond better to the combination of ATM inhibitors and radiotherapy [115], while drug resistance mutations (e.g., ATM pathway attenuation) may be therapeutically exploited via synthetic lethality (ATR inhibition) [116]. These findings indicate that when evaluating treatment strategies, it is necessary to consider the mutation background and the regulation of pathways.

“Diffuse midline glioma” became a frequently used keyword from 2019 to 2024. This indicates that researchers are particularly concerned about this subtype with a high mortality rate. This attention may lead to more investment in innovative experimental designs and the exploration of biomarkers [117]. The frequent occurrence of DMG also indicates that researchers in the field of PGs are currently focusing their studies on addressing the issues of difficult treatment and poor prognosis associated with this condition [118]. Following this trend, research mainly focuses on three directions: one is to target epigenetics [119], the second is to reprogram the immune system, such as using CAR-T [120], and the third is to decipher the tumor microenvironment, like conducting single-cell spatial analysis [118, 121]. Future research should advance multicenter clinical trials, such as the phase III trial similar to ONC201 [122], while integrating multimodal molecular-clinical data streams [118], and establishing a database covering various patient-derived models of PGs [99]. The ultimate goal is to transform keyword popularity into survival rate improvement. Future research also needs to achieve a shorter cycle from basic discoveries to technological breakthroughs and clinical effectiveness through interdisciplinary collaboration, to increase the five-year survival rate of DMG patients.

### Research frontiers

Previous studies have identified many types of PG mutations, but how these variations drive specific clinical phenotypes still requires more research to prove. Future studies may be able to systematically illustrate the complete signaling pathway from genetic mutations to phenotypes in high-risk subgroups (such as DMG patients with H3K27M mutations, etc.) through multi-omics collaboration. This might become a therapeutic window in the future. In the future, deep cooperation with AI can also be carried out, which may enable the discovery of biomarkers that cannot be identified by traditional methods in the big data network, providing data-driven decision support for the optimization of combination therapies (such as immune therapy combinations). If we can further develop machine learning models that combine genetic mutations with clinical data, it might be possible to create more accurate prognostic and predictive models than those based solely on clinical data.

### Limitation

This study has several notable advantages. Firstly, we are the first to employ bibliometric methods to conduct a systematic analysis of research prospects in PGs, thereby providing comprehensive guidance for scholars in this field. This study conducted the first comprehensive bibliometric analysis of 60 years of PGs research, highlighting the significant increase in research investment due to advancements in molecular technology and innovative treatments. The dominant research results in the USA, combined with the strength of Europe and emerging Asian countries, have collectively shaped the global research direction. China has rapidly risen in this study, and its strength should not be underestimated. The shift from traditional therapies to targeted and immunotherapies not only demonstrates the improvement in research methods but also reflects the researchers’ accurate response to the unique biological characteristics of PGs. Future research should focus on promoting multi-omics collaboration, paying particular attention to high-risk subgroups classified by genomics, and finding ways to address the gap in research capabilities between high-income countries and middle- and low-income countries.

## Conclusion

This study presents the first comprehensive bibliometric analysis of six decades of PGs research, highlighting intensified scientific efforts fueled by molecular advancements and therapeutic innovations. Dominant contributions from USA, alongside European and emerging Asian nations, shape the global research trajectory, and China’s rapid rise in this study cannot be underestimated. The evolution from conventional therapies to targeted and immunotherapeutic strategies reflects both methodological refinement and responsiveness to the distinct biological profiles of PGs. Prospective research should emphasize multi-omics collaborations, particularly targeting genomically defined high-risk subgroups, while addressing disparities in research capabilities between high-income and low/middle-income countries. The researchers are exploring the mechanisms linking the molecular expression of tumors with their clinical phenotypes. Such in-depth studies may uncover novel therapeutic windows for PGs. Moreover, the researchers can integrate multi-omics data (such as genomics and transcriptomics) with artificial intelligence to identify new and synergistic biomarkers. Identified research hotspots, specifically DMG and immunotherapy, provide strategic direction for optimizing resources and developing hypotheses.

## Data Availability

The bibliometric analyses were conducted using the Web of Science Core Collection database (Clarivate, https://www.webofscience.com). No additional web-based repositories or accession numbers are required. All search strategies and inclusion criteria are detailed in the Methods section.

## Abbreviations

AI: Artificial intelligence
CAR: Chimeric antigen receptor
CBTTC: Children's Brain Tumor Consortium
CBTN: Children's Brain Tumor Network
CNS: Central Nervous System
DIPG: Diffuse intrinsic pontine glioma
DMG: Diffuse midline glioma
FOP: Fibrodysplasia ossificans progressiva
FUS: Focused ultrasound
GBM: Glioblastoma
HGG: High-grade glioma
IDH: Isocitrate Dehydrogenase
LGG: Low-grade glioma
MAPK: Mitogen-activated protein kinase
NF1: Neurofibromatosis type 1
PA: Pilocytic astrocytoma
PAH: Polycyclic aromatic hydrocarbon
PFS: Progression-free survival
PG: Pediatric glioma
pHGG: Pediatric high-grade glioma
pLGG: Pediatric low-grade glioma
PRC2: Polycomb repressive complex 2
TSC: Tuberous sclerosis complex
WoSCC: Web of Science Core Collection
WHO: World Health Organization
